# The relationship between alexithymia, meaning in life, and quality of life in older breast cancer patients: a cross-sectional study based on variable-centered and person-centered perspectives

**DOI:** 10.3389/fmed.2026.1708876

**Published:** 2026-08-18

**Authors:** Qi Lv, Yawen Deng

**Affiliations:** 1 Surgical Department, Affiliated Hospital of North Sichuan Medical College, Nanchong, Sichuan, China; 2 School of Management, North Sichuan Medical College, Nanchong, Sichuan, China; 3 Key Laboratory of Digital-Intelligent Disease Surveillance and Health Governance, North Sichuan Medical College, Nanchong, Sichuan, China

**Keywords:** alexithymia, breast cancer, latent profile analysis, meaning in life, quality of life

## Abstract

**Background:**

Alexithymia, characterized by difficulties in identifying and expressing emotions, has been linked to diminished quality of life in older breast cancer patients, a vulnerable population facing multifaceted psychosocial challenges. While prior research has explored these associations, investigations have predominantly adopted a variable-centered approach, overlooking the potential statistical mediating role of meaning in life. Moreover, no studies have examined the heterogeneity of alexithymia and meaning in life through a person-centered lens, which may reveal distinct subgroups with different quality of life outcomes among older breast cancer patients.

**Methods:**

A cross-sectional design was employed, recruiting 577 older breast cancer patients from four tertiary grade A hospitals in Sichuan Province, China, between April 2 and May 29, 2025. Participants completed validated self-report measures, including the 20-item Toronto Alexithymia Scale (TAS-20-C), the Meaning in Life Questionnaire (MLQ), the Quality of Life Scale (QOLS), and a demographic questionnaire. PROCESS macro (Model 4) was used to test a cross-sectional statistical mediation model from the variable-centered perspective, while latent profile analysis identified subgroups from the person-centered perspective.

**Results:**

Variable-centered analyses showed that alexithymia was negatively associated with quality of life, and meaning in life statistically accounted for part of this association. Person-centered analyses identified two distinct latent profiles: a high alexithymia—low meaning in life subgroup, characterized by elevated emotional processing deficits and reduced purpose, and a medium alexithymia—medium meaning in life subgroup, exhibiting adaptive emotional regulation and strong existential coherence. The former subgroup reported significantly lower quality of life scores compared to the latter.

**Conclusion:**

By applying meaning construction theory through a dual-perspective framework, this study clarifies patterns of association among alexithymia, meaning in life, and quality of life in older breast cancer patients and highlights the value of person-centered profiling for targeted interventions. These findings offer novel theoretical insights and practical implications, such as tailored psychotherapeutic strategies that may enhance meaning-making and address alexithymia, with the potential to promote quality of life in this population.

## 1 Introduction

Breast cancer is the most common malignant tumor among women worldwide (1, 2). Based on immunohistochemical characteristics, breast cancer can be classified into subtypes such as Luminal A, Luminal B, HER2 overexpression, and triple-negative breast cancer (3, 4). According to the global cancer statistics in 2022, there were 2.308 million new cases of female breast cancer, accounting for 11.6% of all new cancer cases, ranking second in incidence among malignant tumors (5). However, epidemiological data from the United States in 2024 indicated that the incidence and mortality rates of invasive breast cancer were primarily concentrated in women aged 50 and above, with a median age of 62 (6). This underscores the crucial importance of prevention and management strategies for older breast cancer populations (7). Although current breast cancer research primarily focuses on innovations in treatment approaches and improvements in prognosis (8, 9), the severe psychological distress caused by cancer warrants further investigation. This is particularly important for older breast cancer patients, as aging is associated with declines in cognitive ability and a tendency to form biased notions (10). Additionally, the social networks of these patients often shrink, leading to fewer channels for emotional expression, combined with treatment-related trauma, which hinders self-emotional expression and exacerbates alexithymia (11, 12). Alexithymia is a personality-related construct reflecting deficits in emotional awareness and processing (13), and it is commonly conceptualized as comprising three core dimensions: difficulty identifying feelings (DIF), difficulty describing feelings (DDF), and externally oriented thinking (EOT) (14), as operationalised by the 20-item Toronto Alexithymia Scale (TAS-20). These dimensions capture problems in recognising and verbalising internal emotional states and a tendency to focus on external, concrete details rather than subjective experience, which may undermine emotional adjustment during cancer survivorship. Therefore, alexithymia in older breast cancer patients can impair quality of life across multiple dimensions (15). Such impairment may further erode patients’ sense of self-worth and lead to delayed treatment due to inaccurate symptom reporting. Thus, exploring factors associated with the quality of life of older breast cancer patients with alexithymia, and potential pathways for improvement, holds significant practical importance.

In recent years, driven by global population aging and the increasing prevalence of chronic diseases, quality of life has become a key indicator in health research (16). Quality of life encompasses a comprehensive assessment of patients’ physical, psychological, and social functioning and is crucial for evaluating treatment effects and overall health status (17, 18). However, prior studies have primarily emphasized objective factors influencing the quality of life of older breast cancer patients, such as treatment regimens and environmental factors (19, 20), while neglecting the role of subjective psychological factors. This oversight is particularly evident in the older, as subjective psychological states have a more pronounced impact on quality of life (21, 22). Alexithymia manifests as maladaptive behavioral responses in older breast cancer patients under conditions of anxiety, depression, and other psychological states (23). It may weaken emotional regulation capacity, thereby intensifying negative emotions such as anxiety and depression, ultimately reducing quality of life (24). To address these gaps, this study aims to investigate two key questions: (1) Is alexithymia negatively associated with the quality of life of older breast cancer patients? (2) What are the potential pathways linking alexithymia to quality of life, and does meaning in life statistically account for part of this association?

Existing research indicates that enhancing the sense of meaning in life is an effective strategy for improving the quality of life of older breast cancer patients (25, 26). The sense of meaning in life refers to an individual’s perception of their existential value, goal-oriented experiences, and coherent understanding of life events (27). Numerous studies have shown that meaning in life not only help patients achieve psychological balance during illness but also enhance resilience and coping abilities (28, 29). For instance, meaning in life can alleviate death anxiety, improve emotional states, and strengthen treatment adherence, thereby enhancing the quality of life of cancer patients (30). Nevertheless, most previous studies have focused on young and middle-aged breast cancer patients (31, 32), with limited attention to older breast cancer patients. Given the unique psychological and physiological challenges faced by older breast cancer patients, such as social isolation, role changes, and heightened awareness of mortality (33, 34), the role of meaning in life in this population may be even more prominent.

The meaning-construction theory posits that individuals actively construct meaning to cope with life events to maintain psychological balance and promote adaptation (35). For older breast cancer patients, the diagnosis and treatment process often triggers profound reflections on the sense of meaning in life (25, 36). According to this theory, meaning in life can be constructed through two primary pathways: searching for meaning, where individuals actively explore life’s purposes and values; and creating meaning, where individuals reinterpret life events and integrate them into their existing life narratives. Although the process of constructing a sense of meaning in life is inherently subjective and individualized, it plays a positive role in alleviating alexithymia and improving quality of life (37, 38). On one hand, constructing a sense of meaning in life enables older breast cancer patients to reassess the significance of their illness, thereby reducing fear and anxiety (39, 40); on the other hand, enhanced meaning in life can foster psychological resilience, allowing patients to better cope with disease-related life changes (41). In parallel, patients with higher alexithymia tend to report lower perceived meaning/purpose in life and poorer meaning-making when coping with illness-related stressors (42, 43). This pattern is consistent with the notion that impaired emotion awareness and symbolic processing may constrain reflective appraisal and narrative integration of the cancer experience, thereby undermining the presence of meaning. Together, these findings provide empirical support for examining meaning in life as a potential pathway linking alexithymia to quality-of-life outcomes in older breast cancer patients. Consequently, improvements in these patients’ quality of life can strengthen connections with family and society, reduce feelings of loneliness and depression, and establish positive feedback loops. Thus, meaning in life may statistically account for part of the association between alexithymia and quality of life in older breast cancer patients and was therefore examined as a potential statistical mediator in the present study.

To comprehensively investigate these dynamics, this study adopted a dual-perspective approach combining variable-centered and person-centered analyses. Variable-centered methods, which dominate traditional research, focus on the relationships between variables at the population level, such as the direct negative impact of alexithymia on quality of life and the mediating role of meaning in life. This approach provides general insights into average relationships and mechanisms, helping to identify overall patterns and inform widespread intervention measures (44). However, it often overlooks individual heterogeneity, assumes sample homogeneity, and may obscure subgroups with distinct characteristics. In contrast, person-centered methods, such as latent profile analysis (LPA), emphasize within-individual variability and subgroup identification, revealing heterogeneous patterns (45). The LPA offers a person-centered alternative to variable-centered approaches by identifying unobserved subgroups of individuals who share similar patterns across multiple indicators (44). In oncology and psychosomatic research, LPA has been increasingly used to characterise heterogeneity in psychological and symptom presentations and to examine how these latent subgroups differ in clinical outcomes and supportive care needs (46, 47). With respect to breast cancer, LPA has been applied to identify clinically meaningful patient subgroups based on psychosocial or symptom-related indicators (48, 49); however, the joint profiling of alexithymia and meaning in life—and its association with quality-of-life outcomes—remains limited, particularly among older breast cancer patients. Therefore, applying LPA in the present study is expected to provide a more nuanced understanding of patient heterogeneity and to inform more targeted psychosocial interventions.

Therefore, the present study aimed to examine the relationships among alexithymia, meaning in life, and quality of life in older breast cancer patients. Specifically, we (1) tested whether alexithymia was associated with quality of life; (2) examined whether meaning in life statistically accounted for the association between alexithymia and quality of life in a cross-sectional mediation model; and (3) adopted a person-centered approach using latent profile analysis (LPA) to identify distinct subgroups based on patterns of alexithymia and meaning in life and to compare subgroup differences in quality-of-life outcomes. These objectives were intended to clarify patterns of association and patient heterogeneity, rather than to establish causal or temporal relationships.

## 2 Method

### 2.1 Participants

This study aimed to explore how alexithymia impacts the quality of life in breast cancer patients. The inclusion criteria for the target participants were as follows: (1) clinically diagnosed with breast cancer; (2) possession of normal language communication abilities; (3) absence of significant cognitive impairment; and (4) age greater than 60 years. Cognitive impairment was assessed using the Mini-Mental State Examination (MMSE), with a total score below 23 as the standard for exclusion.

The age is set at 60 years old, which is mainly due to the fact that the legal retirement age of most working women in China is between 50 and 55 years old, and 60 years old is officially recognized as the elderly status in national policies, social welfare reminders and the vast majority of gerontology (50, 51). Subsequently, the International Society for Geriatric Oncology and major geriatric oncology guidelines generally recommended ≥65 or ≥70 as a threshold for elderly cancer patients. However, SIOG itself has recognized that the definition of “older adults” is contextual and should be considered in conjunction with national demographics, biological aging, and specific research questions (52, 53). At the same time, in recent years, a large number of scholars have begun to use elderly breast cancer patients ≥60 years old as the inclusion criteria. For example, in Choi et al. (54), Li et al. (55), and Mao et al. (56), the age of elderly breast cancer survivors ≥60 years old was used as one of the criteria. The age criterion of greater than 60 years was based on World Health Organization standards, which have been widely applied and recognized (57, 58). Breast cancer diagnosis was verified by reviewing participants’ medical records at the recruiting hospital (e.g., pathology and/or physician documentation). Patients with metastatic disease (stage IV) were not excluded; thus, both non-metastatic and metastatic breast cancer patients were eligible. Metastatic status was extracted from medical records and reported in the clinical characteristics.

The study was approved by the Academic Ethics Committee of North Sichuan Medical College (No.:2025020). Participants were recruited using convenience sampling from four hospitals. To minimise selection bias across sites, the same eligibility criteria and recruitment procedure were applied in all hospitals. Data collection took place from April 2, 2025, to May 29, 2025, across four Grade A tertiary hospitals in Sichuan Province, China. Initially, we contacted the directors of the thyroid and breast surgery departments in the four hospitals, presented and explained the research protocol, and obtained their approval. Subsequently, data collection was conducted in the inpatient and outpatient departments of the thyroid and breast surgery units in the four hospitals. Patients were provided with detailed information about the study’s purpose, risks, and benefits, and they signed an informed consent form before completing questionnaires on alexithymia, meaning in life, death anxiety, and demographic information.

A total of 619 questionnaires were distributed, and 601 questionnaires were returned. During the data cleaning phase, 24 questionnaires with highly consistent responses or incomplete answers were excluded. The final effective sample size was 577, with an effective rate of 96.16%. Specifically, 39 male patients (6.8%) and 538 female patients (93.2%) participated. The age distribution was primarily concentrated in the 60–65 age group, with 274 patients (47.5%). The cancer stages were mainly concentrated in stage III (38.6%) and stage IV (39.5%). Detailed demographic information is provided in Appendix A. Although male patients were included in the study to reflect the full clinical population encountered in practice, they represented a small proportion of the sample. Therefore, sex-stratified latent profile analyses were not conducted due to limited statistical power and concerns regarding the stability of class estimation.

### 2.2 Research tools

#### 2.2.1 Alexithymia scale

The alexithymia scale was adapted from the 20-item Toronto Alexithymia Scale (TAS-20) developed by Bagby et al. (59). The scale was cross-culturally validated in a Chinese adolescent population by Ling et al. (60). In previous studies, the scale has been widely used to assess emotional difficulties, alexithymia, and emotional suppression in breast cancer patients (61, 62). A 6-point Likert scale was used for scoring, ranging from “1 = strongly disagree” to “6 = strongly agree,” with higher scores indicating more severe alexithymia. The Cronbach’s *α* for the alexithymia scale in this study was 0.939. To further verify the validity of the scale in older patients with breast cancer, we conducted a confirmatory factor analysis of the scale. The results showed that the scale had good construct validity (GFI = 0.885, AGFI = 0.836, RMSEA = 0.078, CFI = 0.927, TLI = 0.906, *χ*^2^/df = 4.492).

#### 2.2.2 Meaning in questionnaire scale

The meaning in life questionnaire (MLQ) was based on the 10-item scale developed by Steger et al. (63), which includes two subscales: search for meaning and presence of meaning. The scale was translated and validated for cultural adaptability and reliability in a Chinese population by (64) and has been widely applied to Chinese cancer patients. A 6-point Likert scale was used, ranging from “1 = strongly disagree” to “6 = strongly agree.” Higher scores indicated a greater sense of meaning in life. The Cronbach’s *α* coefficient for the meaning in life scale in this study was 0.787, which was acceptable. In the PROCESS macro (Model 4), meaning in life was treated as a single latent construct representing the overall level of existential meaning. Although the MLQ includes two subdimensions (presence of meaning and search for meaning), the total score was used in PROCESS macro (Model 4) to capture the global psychological resource of meaning in life in the context of illness adaptation. In contrast, the LPA was conducted using item-level indicators of the MLQ. Using item-level responses allowed for a more fine-grained identification of heterogeneity in participants’ meaning-related experiences, which is consistent with the person-centered nature of LPA.

#### 2.2.3 Quality of life scale

The quality of life scale (QOLS) was based on the 16-item instrument developed by Burckhardt and Anderson (65), which includes five dimensions and a stand-alone item: “Physical and material wellbeing,” “Relationships with others,” “Social, community, and civic activities,” “Personal development and achievement,” and “Recreation.” The scale has been widely applied to Chinese patient populations (66), with validated cultural adaptability. A 6-point Likert scale was used, ranging from “1 = strongly disagree” to “6 = strongly agree.” Higher scores indicated better quality of life. The Cronbach’s *α* coefficient for the quality of life scale in this study was 0.855, indicating good internal consistency.

### 2.3 Data analysis

All questionnaire data were double-entered into Microsoft Excel and cross-checked by two researchers. Statistical analyses were conducted using SPSS 27.0 and Mplus 8.0. First, common method bias was examined using Harman’s single-factor test, followed by descriptive statistics and Pearson correlation analyses. Among them, we used the skewness and kurtosis of the core study variables to assess the normality of the study data.

Second, a cross-sectional mediation analysis was conducted in SPSS using the PROCESS macro (Model 4), with alexithymia (TAS-20) as the independent variable, meaning in life (MLQ) as the mediator, and quality of life (QOLS) as the dependent variable; gender, place of residence, and cancer stage were included as covariates. In line with recommendations for cross-sectional mediation models, the indirect effect was interpreted as statistical mediation rather than evidence of causal mediation. Indirect effects were evaluated using bootstrapping with 5,000 resamples and bias-corrected 95% confidence intervals.

Third, the LPA was performed in Mplus 8.0 using item-level indicators from TAS-20 and MLQ. Model fit was compared across 1–5 class solutions using AIC, BIC, sample-size adjusted BIC (aBIC), entropy, the Lo–Mendell–Rubin adjusted likelihood ratio test (LMR–LRT), and the bootstrapped likelihood ratio test (BLRT). To enhance transparency and facilitate interpretation of the latent profiles, class-specific means for all LPA indicators in the selected model were reported in tabular form in addition to the profile plot.

Finally, differences in quality of life across the optimal latent profiles were examined using one-way ANOVA in SPSS. Prior to the ANOVA, the assumptions of normality and homogeneity of variance were assessed. Levene’s test was employed to evaluate variance equality, and Welch’s robust test was applied as a supplementary analysis when the homogeneity assumption was violated.

## 3 Results

### 3.1 Common method bias

To preliminarily assess the possibility of common method bias, Harman’s single-factor test was conducted using exploratory factor analysis on all measurement items. The results showed that, in the unrotated solution, the first factor accounted for 26.495% of the total variance, which was below the commonly used threshold of 40%. This finding did not indicate a dominant single-factor structure. However, because Harman’s single-factor test is only a preliminary diagnostic method, common method bias cannot be completely ruled out.

### 3.2 Descriptive statistics and correlation analysis

We performed descriptive statistics and correlation analysis on alexithymia, meaning in life, and quality of life. The results showed the following: alexithymia was significantly negatively correlated with quality of life (*r* = −0.365, *p* < 0.001); alexithymia was significantly negatively correlated with meaning in life (*r* = −0.190, *p* < 0.001); and meaning in life was significantly positively correlated with quality of life (*r* = 0.501, *p* < 0.001). Specific results are detailed in Table 1.

**Table 1:** Descriptive statistics and correlation analysis results.

Variable	*M*	SD	Skewness	Kurtosis	1	2	3
1. Alexithymia	3.221	0.921	1.410	1.762	1		
2. Meaning in life	3.089	0.649	−0.200	3.713	−0.190^***^	1	
3. Quality of life	2.945	0.569	−1.322	2.916	−0.365^***^	0.501^***^	1

^***^p < 0.001.

The skewness values of the core variables ranged from −1.322 to 1.410, and the kurtosis values ranged from 1.762 to 3.713, which met the criteria proposed by Kline (67) (skewness < |3|, kurtosis < |8|). Therefore, the core study variable data in this study conformed to an approximately normal distribution.

### 3.3 Cross-sectional statistical mediation analysis

To examine whether meaning in life statistically accounted for the association between alexithymia and quality of life, we specified a cross-sectional mediation model in which meaning in life was entered as the mediator, alexithymia as the independent variable, and quality of life as the dependent variable, with gender, residence, and breast cancer stage included as covariates. The results indicated that: (1) alexithymia was significantly negatively associated with meaning in life (*β* = −0.132, *p* < 0.001, 95% CI = [−0.189, −0.075]); (2) alexithymia was significantly negatively associated with quality of life (*β* = −0.166, *p* < 0.001, 95% CI = [−0.208, −0.124]); and (3) meaning in life was significantly positively associated with quality of life (*β* = 0.391, *p* < 0.001, 95% CI = [0.331, 0.451]) (Table 2).

**Table 2:** Regression coefficients for the mediating effect of meaning in life.

Regression equation		Overall fit index			Significance of regression coefficient		
Outcome variables	Predictive variables	*R*	*R* ^2^	*F*	*β*	*t*	95%CI
Meaning in life	Alexithymia	0.207	0.043	6.431^***^	−0.132	−4.549^***^	[−0.189, −0.075]
Quality of life	Alexithymia	0.583	0.340	58.833^***^	−0.166	−7.708^***^	[−0.208, −0.124]
	Meaning in life				0.391	12.837^***^	[0.331, 0.451]

^*^p < 0.05; ^**^p < 0.01; ^***^p < 0.001.

**Figure 1:**
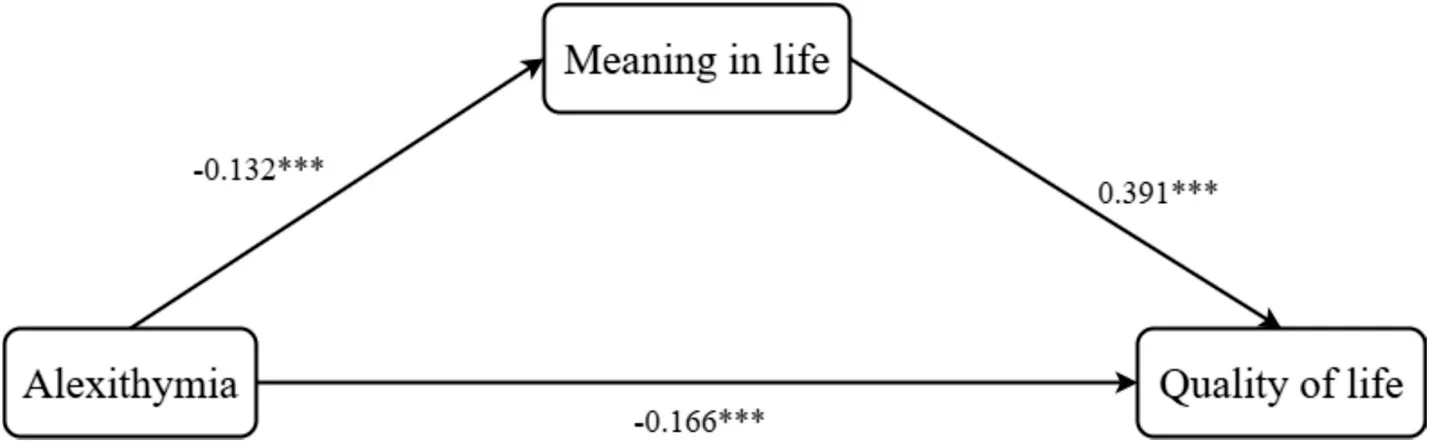
Table of path coefficients for the mediating role of meaning in life. ^***^p < 0.001.

Total and mediated effects. As shown in Figure 1 and Table 3, the total effect of alexithymia on quality of life in breast cancer patients was −0.217. Of this, 24% was mediated by meaning in life. This included a significant direct effect of alexithymia on quality of life (*β* = −0.166, SE = 0.022, 95% CI = [−0.208, −0.124]). Additionally, there was a significant partial mediating effect through meaning in life (*β* = −0.052, SE = 0.027, 95% CI = [−0.113, −0.007]).

**Table 3:** Decomposition of total, direct, and indirect associations in the cross-sectional mediation model.

Type of effect	*β*	SE	LLCI	ULCI	Percentage
Total effect	−0.217	0.024	−0.265	−0.170	
Direct effect	−0.166	0.022	−0.208	−0.124	76%
Indirect effect	−0.052	0.027	−0.113	−0.007	24%

Direct effect, Alexithymia-Quality of life; Indirect effect, Alexithymia-Meaning in life-Quality of life.

### 3.4 Latent profile analysis

This study used Mplus 8 software to perform latent profile analysis on the measurement items of alexithymia and meaning in life in breast cancer patients, constructing 1–5 latent profile models to evaluate and determine the optimal fitting model. Model selection was based on a combination of statistical fit and substantive interpretability. Specifically, we primarily considered information criteria (AIC, BIC, and sample-size adjusted BIC [aBIC]; lower values indicate better fit) and likelihood ratio tests (Lo–Mendell–Rubin adjusted LRT [LMR–LRT] and the bootstrapped likelihood ratio test [BLRT]) to determine whether a k-class solution significantly improved fit over the (*k* − 1)-class solution. Entropy was treated as an auxiliary indicator of classification precision rather than a sole decision criterion. Practical considerations, including adequate class size and conceptual coherence of the profile patterns, were also taken into account.

The fit indices for each model are summarized in Table 4. While the AIC, BIC, and aBIC values improved with the increasing number of latent classes, the entropy value of the 2-class model (Entropy = 0.998) was higher compared to the 3-class model (Entropy = 0.899), indicating that the 2-class model had clearer classification. Compared to the 4- and 5-class models, the LMR and BLRT values for the 2-class model were statistically significant (*p* < 0.001). Average posterior probabilities for most likely class membership were 1.000 for Class 1 and 0.998 for Class 2, indicating clear class separation. Therefore, the 2-class latent profile model was selected as it was more scientifically robust and clear.

**Table 4:** Fit indices for the 5-class latent profile models.

Profile	AIC	BIC	aBIC	Entropy	LMR (*p*)	BLRT (*p*)	Smallest proportion per class
1	56,960.526	57,221.996	57,031.520				
2	52,136.570	52,533.134	52,244.246	0.998	<0.001	<0.001	0.861/0.139
3	51,077.439	51,609.096	51,221.795	0.899	0.009	<0.001	0.380/0.487/0.133
4	50,284.882	50,951.632	50,465.919	0.925	0.533	<0.001	0.386/0.454/0.073/0.087
5	49,477.801	50,279.644	49,695.519	0.947	0.154	<0.001	0.079/0.370/0.071/0.413/0.067

AIC, Akaike Information Criterion; BIC, Bayesian Information Criterion; aBIC, Sample-Size Adjusted BIC; LMR, Lo–Mendell–Rubin Adjusted LRT Test; BLRT, Bootstrapped Likelihood Ratio Test.

Although the 3-class solution also showed acceptable statistical fit, inspection of the profile patterns indicated that the additional class primarily reflected a quantitative split of the moderate group rather than a qualitatively distinct subgroup with clear clinical meaning. In other words, the 3-class model did not yield a new profile characterized by a unique combination of alexithymia and meaning-in-life indicators; instead, it mainly separated participants along severity levels with substantial overlap in the overall response pattern. Similarly, the 4-class and 5-class solutions produced smaller classes that were less stable and less clinically interpretable, and some of these classes appeared to represent further fragmentation of existing profiles rather than clinically actionable subgroups. Because latent profile selection should consider not only statistical fit but also parsimony, class size, stability, and clinical interpretability, the 2-class model was retained as the most appropriate solution for the present sample. From a clinical perspective, the 2-class solution provided the clearest distinction between a relatively lower-risk subgroup and a higher-risk psychosocial subgroup, which may be more readily translated into screening and supportive care strategies in older breast cancer patients.

**Figure 2:**
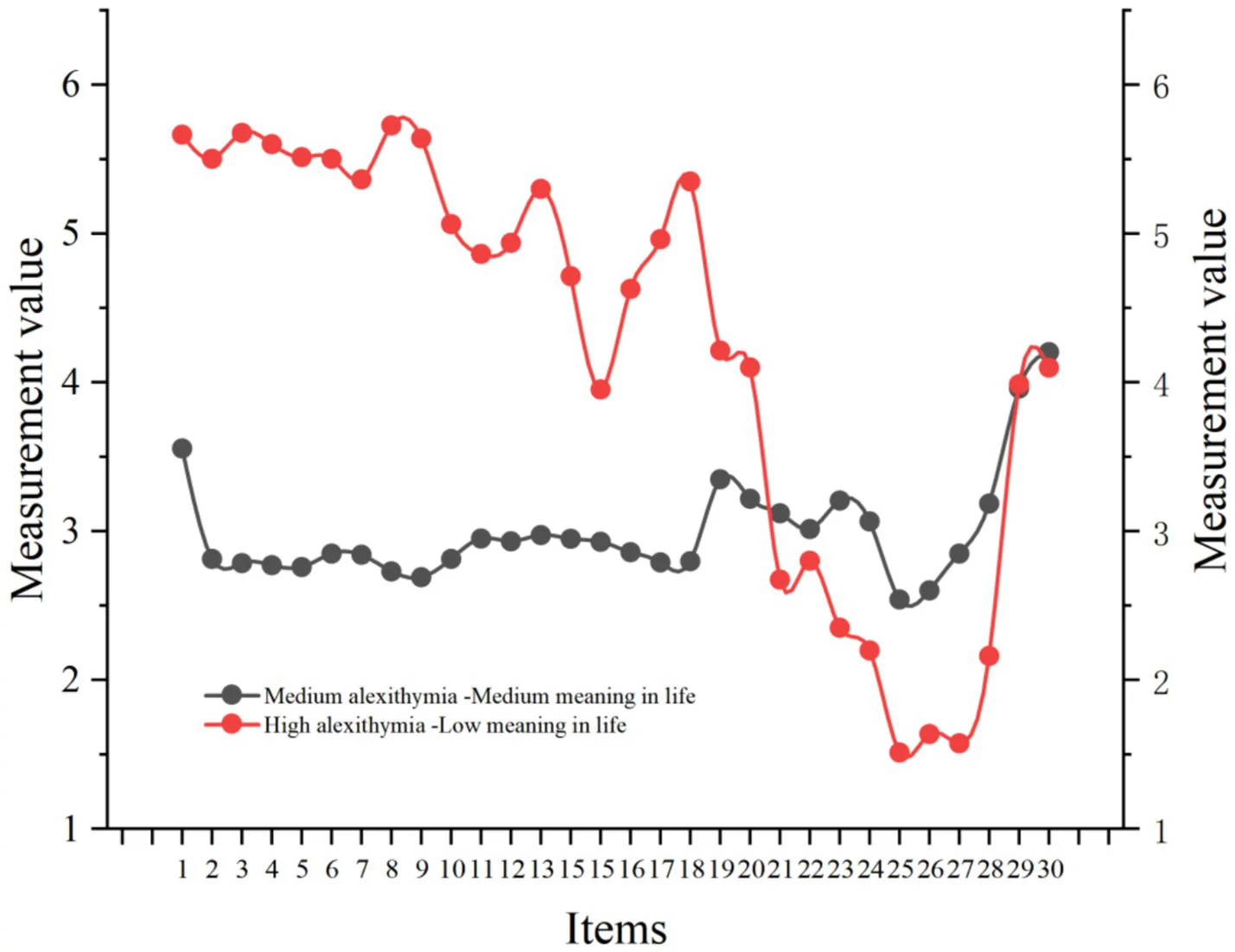
Latent subgroups of alexithymia and meaning in life in breast cancer patients.

Based on the analysis in Table 4, we used Origin 2021 software to plot the latent profiles of the 2-class model, as shown in Figure 2. The results indicated: (1) the first latent subgroup was the moderate alexithymia–moderate meaning in life group, accounting for 86.1% of the sample (*N* = 497). This group represented breast cancer patients who had some difficulty in emotional recognition and expression but maintained a moderate level of meaning in life. (2) The second latent subgroup was the high alexithymia–low meaning in life group, accounting for approximately 13.9% of the sample (*N* = 80). This group represented breast cancer patients with significantly impaired emotional recognition and expression abilities and low levels of meaning in life.

### 3.5 Single-factor ANOVA of latent subgroups and quality of life

To further examine the differences in quality of life among the latent subgroups, we used the latent subgroups of alexithymia and meaning in life as independent variables and quality of life as the dependent variable, performing a single-factor ANOVA. Prior to conducting the ANOVA, key statistical assumptions were evaluated. Regarding homogeneity of variance, Levene’s test based on the mean indicated a significant violation of the equal variance assumption (*F*(1, 575) = 73.381, *p* < 0.001). Consequently, in addition to the standard ANOVA, Welch’s robust test for equality of means was conducted to account for this heteroscedasticity.

The standard ANOVA results showed that the quality of life in the moderate alexithymia–moderate meaning in life group (*M* = 3.045, SD = 0.448) was significantly higher than that in the high alexithymia–low meaning in life group (*M* = 2.341, SD = 0.821), with a significant overall effect (*F*(1, 575) = 128.376, *p* < 0.001, *η*^2^ = 0.183). This finding was corroborated by Welch’s robust test (Welch’s *F*(1, 86.735) = 56.123, *p* < 0.001), confirming that the significant difference in quality of life between the two latent subgroups was not an artifact of unequal variances. The medium-to-large effect size (*η*^2^ = 0.183) further underscores the practical significance of this subgroup distinction, indicating that approximately 18.3% of the variance in quality of life was attributable to latent subgroup membership.

The differences in demographic information among the potential subgroups are presented in Appendix A. Meanwhile, the results of the multiple Logistic regression analysis of this study are presented in Appendix A.

## 4 Discussion

### 4.1 Latent profile analysis

The results of the latent profile analysis in this study revealed heterogeneity among older breast cancer patients in terms of alexithymia and meaning in life, identifying two distinct latent subgroups: a high alexithymia–low meaning in life group and a moderate alexithymia–moderate meaning in life group. The former is characterized by significant deficits in emotional identification and expression, as well as a lower perception of existential value and life coherence. The latter exhibits relatively moderate emotional processing abilities and maintains a certain level of meaning in life. This classification not only highlights the critical role of individual differences in psychological adaptation to cancer but also aligns with the results of the subsequent single-factor ANOVA, which showed that the high-risk group had significantly lower quality of life scores compared to the low-risk group, particularly in the domains of physical, emotional, and social functioning.

Patients in the “high alexithymia–low meaning in life” group may face compound psychosocial burdens associated with the co-occurrence of emotional processing deficits and existential deprivation. This interpretation is supported by converging empirical evidence from oncology and geriatric populations. Regarding the alexithymia component, a growing body of research has demonstrated that alexithymia is prevalent among cancer patients and is consistently associated with maladaptive coping strategies—such as avoidance and emotional suppression—that that have been linked to greater psychological distress and poorer health outcomes (68, 69). Specifically in breast cancer populations, Boinon et al. (61) found that alexithymia significantly impaired social sharing after surgery, which in turn amplified emotional distress and reduced perceived social support. In older adults, alexithymia has been linked to heightened vulnerability to depression and anxiety, possibly in part due to age-related declines in cognitive flexibility and social network shrinkage that may reduce opportunities for emotional processing and expression (70). These findings suggest that older breast cancer patients with high alexithymia may be doubly disadvantaged, as both disease-related trauma and aging processes may converge to constrain their emotional regulation capacity.

Regarding the meaning in life component, empirical evidence from oncology settings has consistently shown that low meaning in life is a robust predictor of poor psychological adjustment and diminished quality of life in cancer patients. Nilsen et al. (25) reported that among older long-term breast cancer survivors, reduced meaning-making was significantly associated with lower psychological adjustment and poorer quality of life, underscoring the particular salience of existential concerns in this demographic. Park (71) proposed within the meaning-making model that when individuals fail to reconcile situational meaning (e.g., the cancer diagnosis) with global meaning (e.g., pre-existing beliefs about justice and purpose), they experience sustained psychological distress—a process that may be particularly constrained in patients with concurrent alexithymia, who may lack the emotional awareness necessary to engage in effective meaning-making.

Critically, the coexistence of high alexithymia and low meaning in life in this subgroup may reflect a pattern of co-occurring psychosocial vulnerability rather than a single underlying process. According to meaning-construction theory (72), the process of meaning reconstruction following significant life events requires both cognitive reappraisal and emotional engagement. Alexithymia may constrain patients’ capacity to engage in meaning reconstruction, potentially contributing to a pattern in which emotional processing deficits hinder meaning-making, and the resulting absence of meaning may further reinforce emotional dysregulation and existential distress. Stanton et al. (73) demonstrated that emotional processing and emotional expression serve as critical facilitators of psychological adjustment in breast cancer patients, and that their absence predicts deteriorated health outcomes. In older populations specifically, Krause (74) found that meaning in life served as a buffer against the deleterious effects of emotional suppression on wellbeing. Thus, this subgroup may represent a highly vulnerable psychological phenotype characterized by a pattern of co-occurring emotional processing deficits and existential deprivation that may be mutually reinforcing.

In contrast, patients in the “moderate alexithymia–moderate meaning in life” group may retain some emotional awareness and benefit from a certain level of meaning in life, which could aid in adapting to cancer-related challenges. Although they are not entirely free from psychological distress, this subgroup may possess sufficient internal and external resources to maintain a relatively stable quality of life. This characteristic aligns with previous research (75), which indicates that even moderate levels of meaning in life have been associated with psychological resilience, even when emotional functioning is partially impaired.

From the perspective of meaning-construction theory, the presence of meaning and the search for meaning represent complementary aspects of individuals’ efforts to understand and integrate stressful life events. In older adults coping with cancer, the search for meaning may reflect ongoing attempts to reinterpret illness experiences, whereas the presence of meaning reflects the extent to which individuals have successfully integrated these experiences into their life narrative. In the present study, meaning in life was conceptualized as an overall existential resource in the variable-centered analysis, while item-level responses were used in the person-centered analysis to capture potential heterogeneity in meaning-related experiences among older breast cancer patients.

From a clinical practice perspective, the results of this study provide actionable guidance for stratified intervention strategies. Although the high alexithymia–low meaning in life group represents a small proportion of the sample, the degree to which quality of life was lower in this group is significant, suggesting that clinicians should prioritize screening for such individuals. For example, using simple alexithymia screening tools in combination with assessments of meaning presence, high-risk patients can be identified early in diagnosis and targeted interventions can be implemented, such as meaning-oriented group therapy, to help them shift from a “victim” narrative to a “survivor” framework. For example, in Breitbart et al. (76), individual meaning-centered psychotherapy showed sustained improvements in meaning, quality of life, and symptom burden compared with therapeutic massage, and the effects remained stable at two-month follow-up. For the moderate alexithymia–moderate meaning in life group, interventions can focus on reinforcing supportive measures, such as family-based emotional expression training, to prevent potential deterioration. Manne et al. (77) developed and evaluated an Intimacy-Enhancing Psychological Intervention for early-stage breast cancer patients and their partners, which demonstrated significant improvements in relationship intimacy, emotional expression, and psychological adjustment. Additionally, in the cultural context of older breast cancer patients in China, interventions should incorporate local elements, such as the construction of filial meaning, to enhance compliance and efficacy. Ho et al. (78) showed that family-based meaning-making interventions that incorporated culturally specific values significantly enhanced psychological adjustment among Chinese cancer patients. These findings not only improve the efficiency of resource allocation but also align with the shift in modern nursing from homogenization to personalized care.

### 4.2 Variable-centered perspective

#### 4.2.1 Theoretical implications

The study found that alexithymia in older breast cancer patients was significantly negatively associated with their quality of life. In recent years, the incidence of breast cancer among the older has been increasing, and older breast cancer patients may experience a heightened burden of illness due to age-related physiological and psychosocial factors (53). Studies have shown that, compared to younger patients, older breast cancer patients are more likely to face reduced social support due to factors such as loneliness, the death of a spouse, or children moving away from home (79). These factors have been linked to exacerbated psychological health issues, particularly depression and anxiety-related alexithymia (80). However, most previous research has focused on the biomedical characteristics and treatment outcomes of older breast cancer patients (81), with little attention paid to alexithymia. To address this gap, this study used a cross-sectional design to confirm that alexithymia in older breast cancer patients is significantly negatively associated with quality of life. Specifically, alexithymia was significantly associated with reduce functional status, which may affect daily activities and social interactions. For instance, prior research has suggested that depression is associated withloss of interest, reduced energy, and sleep disturbances, which may make it difficult for patients to perform daily self-care and family responsibilities (82). Furthermore, psychological health issues may be associated with weakened patients’ adherence to cancer treatment, potentially contributing to poor treatment outcomes and prognosis (83). Studies have shown that breast cancer patients with depressive symptoms are more likely to discontinue radiotherapy or chemotherapy (84). Additionally, alexithymia has been associated with more symptoms such as pain and fatigue, further reducing physical discomfort and overall quality of life (85). Therefore, addressing alexithymia in older breast cancer patients may be important for promoting their quality of life. This finding highlights the high prevalence of psychological health issues in older breast cancer patients and their significant associations with quality of life, urging clinicians and researchers to pay greater attention to the psychological health of this special population.

This study also found that meaning in life statistically accounted for part of the association between alexithymia and quality of life in older breast cancer patients. From a theoretical perspective, it is plausible that when patients achieve self-acceptance and better cope with the physical and mental changes brought about by the disease, they may reduce excessive anxiety about “defects,” diminish self-denial, and improve emotional recognition abilities, which could in turn be associated with a reduction in the negative correlates of alexithymia, such as emotional accumulation, reduced happiness, marital crises, anxiety, depression, and communication difficulties (86, 87). Such improvements may also be related to enhance patients’ self-efficacy and health management consciousness. Similarly, by strengthening self-worth and reconceptualizing meaning in life, patients may be better motivated strengthen their motivation to participate in treatment and social activities, potentially reducing feelings of isolation and promoting cooperating with treatment, adhering to rehabilitation exercises, and improvements in emotional expression difficulties that have been linked to avoidance of social activities and loss of social support resources (88, 89). Additionally, a reduction in communication barriers with healthcare providers may help mitigate the challenges of individualized treatment (90). Through these pathways, patients may be better positioned to transform disease challenges into opportunities for self-healing, enhance their confidence in overcoming the psychological suffering caused by alexithymia, and ultimately experience improved their quality of life (91). These findings not only deepen theoretical research at the intersection of psychology and oncology but also provide important theoretical support for clinical practice. Meaning in life, as a process of understanding and assigning value to life (92), has garnered significant attention in the field of mental health but has been relatively understudied in older breast cancer populations. By showing that meaning in life was related to both alexithymia and quality of life, this study provides new empirical support for the concept of meaning in life and offers theoretical considerations regarding the relationship between emotional expression and health outcomes.

Finally, this study provides empirical support for meaning-construction theory while refining its application within the context of geriatric oncology. Meaning-construction theory posits that individuals adapt to major life stressors by reconstructing meaning through cognitive and emotional processing (93). However, older breast cancer patients often experience pronounced difficulties in emotional identification and expression due to both age-related psychosocial changes and illness-related distress (94). These emotional processing deficits may hinder the ability to reinterpret illness experiences and integrate them into a coherent life narrative (95). The present findings are consistent with the possibility that alexithymia may constrain this adaptive meaning-construction process by limiting emotional awareness and expression, which may in turn be associated with a reduced capacity to derive meaning from the cancer experience. Conversely, a stronger sense of meaning in life may serve as a protective factor in the association between alexithymia and quality of life, highlighting the importance of meaning reconstruction as a psychological adaptation pathway. Importantly, the results are consistent with the theoretical proposition that emotional expression may be a key factor facilitating meaning construction: it is plausible that when patients are better able to recognize and articulate their emotional experiences, they may more effectively reframe illness-related adversity and maintain psychological equilibrium. By identifying the statistical mediating role of meaning in life in the association between alexithymia and quality of life, this study extends meaning-construction theory to an understudied population—older breast cancer patients. It further highlights emotional expression as a potentially important adaptive factor within the meaning-construction process, offering a more nuanced understanding of how emotional processing may relate to existential adaptation and wellbeing in later-life cancer contexts.

#### 4.2.2 Practical implications

The finding that meaning in life serves as a significant statistical mediator in the association between alexithymia and quality of life of older breast cancer patients provides important theoretical and practical guidance for clinical practice. Older breast cancer patients, as a special subgroup, face age-related declines in physical function, changes in social roles, and deeper existential challenges, such as heightened awareness of mortality. These factors may predispose older patients to greater alexithymia and lower quality of life. However, the study findings are consistent with the notion that meaning in life is associated with psychological health states, including lower death anxiety and greater psychological resilience. In light of these observed associations, clinicians may consider implementing evidence-based psychological interventions that target meaning reconstruction and emotional processing. Meaning-centered interventions, which aim to help patients rediscover life purpose and existential value in the context of illness, have demonstrated effectiveness in improving psychological wellbeing and quality of life among cancer patients in randomized clinical trials and meta-analyses (26, 76). In addition, cognitive-behavioral therapy (CBT) has been widely shown to reduce psychological distress, anxiety, and depressive symptoms in oncology populations, thereby contributing to improved adjustment and quality of life (96–98). Through these interventions, patients may learn to reinterpret illness experiences, enhance emotional awareness and expression, and develop more adaptive coping strategies. Such approaches are particularly relevant for older breast cancer patients who experience difficulties in emotional identification and expression, as improving emotional processing may be associated with enhanced meaning reconstruction and may in turn contribute to better quality of life.

In clinical practice, older patients often face multiple chronic diseases and complex treatment regimens, making them more dependent on family support and social resources during treatment. However, meaning in life, as an internal psychological resource, can help patients find inner strength and motivation when facing the dual challenges of disease and aging. Integrating meaning-centered approaches within multidisciplinary cancer care may further enhance patient support. Conventional oncology care primarily focuses on medical treatment and symptom management, whereas meaning-centered approaches address existential concerns such as life purpose, personal values, and the search for meaning in the face of illness. By helping patients reinterpret their illness experience within a broader life narrative, meaning-centered care may complement biomedical treatment and promote psychological resilience. Effective implementation of such approaches often requires collaboration among healthcare professionals, including oncologists, nurses, psychologists, and social workers, who can jointly address patients’ physical, emotional, and existential needs. Within this collaborative framework, meaning-centered strategies may serve as a bridge connecting medical treatment with psychosocial and existential support, thereby contributing to a more holistic model of cancer care for older patients.

A core goal of nursing practice is to improve patients’ quality of life, and meaning in life, as a key psychological resource, can help patients better adapt to disease-related changes and find meaning and value in life. For older breast cancer patients, nurses can use patient-centered care models, combined with individualized care plans, to help patients explore and reconstruct meaning in life. Nurses can listen to patients’ experiences and feelings, helping them reflect on past achievements and unfulfilled goals to find meaning in their current lives. By focusing on meaning in life, nursing practice may contribute to improve psychological health and help patients find inner peace and satisfaction amid disease and aging, thereby supporting high-quality end-of-life care.

### 4.3 Limitations and future research directions

Despite shedding light on the complex relationships between alexithymia, meaning in life, and quality of life in older breast cancer patients using a dual-perspective analytical framework, this study has several limitations, which point to clear directions for future research. First, the study employed a cross-sectional design, which, while able to reveal associations and mediating mechanisms, cannot establish causal relationships. The dynamic interactions between alexithymia, meaning in life, and quality of life may evolve over time, particularly during disease progression, changes in treatment stages, or the implementation of psychological interventions. Future research should adopt longitudinal designs or multiple time-point tracking to capture the temporal trajectories of these variables and apply cross-lagged models or growth curve models to test causal pathways. Additionally, randomized controlled trials could further validate whether meaning-oriented psychological interventions alleviate alexithymia by enhancing meaning in life and ultimately improve quality of life. Such studies could strengthen the causal inferential power of theoretical mechanisms and provide more time-sensitive intervention strategies for clinical practice.

Second, the study’s sample was drawn from four Grade A tertiary hospitals in Sichuan Province, China, which limits the representativeness of the findings in terms of regional and cultural diversity. China’s vast regional variations in medical resources, cultural backgrounds, and social support systems may influence the expression of alexithymia, the mechanisms of meaning in life construction, and the evaluation standards for quality of life. Future research should conduct multicenter, cross-regional, or even cross-cultural comparative studies to test the generalizability and differences of the current model in different healthcare environments and cultural contexts. Furthermore, the proportion of male breast cancer patients in the sample was relatively low, and future studies should intentionally expand the sample size of male or non-binary gender patients to explore the moderating role of gender in the relationship between alexithymia and meaning in life, thereby providing a more inclusive theoretical framework and practical guidance.

Third, the study primarily relied on self-report questionnaires for data collection, and while the tools used had good reliability and validity, there is still a risk of common method bias and social desirability effects. In particular, since alexithymia involves inherent difficulties in emotional identification, self-reports may not fully capture its complex manifestations. Although Harman’s single-factor test was conducted as a preliminary assessment, this approach is limited and cannot fully rule out shared method variance. Future research should employ multimethod assessment strategies, such as behavioral observations, clinical interviews, physiological indicators (e.g., heart rate variability, cortisol levels), or neuroimaging data, to more comprehensively and objectively assess emotional processing deficits. Additionally, third-party reports (e.g., from healthcare providers or family members) could be used to cross-validate quality of life outcomes. Furthermore, qualitative studies could provide deeper insights into how older breast cancer patients subjectively experience and construct meaning, offering richer theoretical hypotheses and contextual interpretations for quantitative research.

Another limitation relates to the clinical composition of the sample. A relatively high proportion of participants had advanced-stage disease, which may differ from the stage distribution typically reported in many cohorts of older breast cancer patients. Advanced disease may be associated with greater symptom burden, treatment intensity, uncertainty about prognosis, and functional impairment, all of which may influence psychological variables such as alexithymia and meaning in life. Therefore, the latent profile distribution and related associations observed in this study may partly reflect the psychological impact of greater disease burden. This should be considered when interpreting the findings, and the generalizability of the results to older breast cancer populations with predominantly early-stage disease may be limited.

Although male patients accounted for a small proportion of the sample, previous research suggests that men with breast cancer may experience distinct psychosocial challenges, including differences in illness perception, stigma, social support, and emotional expression. Because the number of male participants in the present study was limited, sex-specific analyses were not conducted, and all analyses were performed on the pooled sample to preserve statistical power and model stability. As a result, the identified latent profiles may primarily reflect the overall sample pattern and may not fully capture sex-specific psychosocial differences. Future studies with larger samples of male breast cancer patients are needed to examine whether the profile structure and associated factors differ by sex.

Finally, the construct of meaning in life may involve cultural specificity. The interpretation, expression, and psychological function of meaning in life may vary across sociocultural contexts. Therefore, caution is warranted when generalizing the present findings beyond the cultural setting of this study, and future research should further examine the cross-cultural applicability of these relationships. The present study did not include potentially important treatment-related variables, such as active chemotherapy, endocrine therapy, or other clinical treatment characteristics. These factors may influence patients’ psychological experiences and could serve as important covariates in future studies. Although latent profiles were identified in this study, no external validation was conducted. As a result, the robustness and practical significance of the latent profiles require further examination. Future studies should attempt to validate these profiles using external clinical, behavioral, or psychosocial indicators, as well as replication in independent samples.

## 5 Conclusion

This study, through a dual-perspective analytical framework, elucidated the complex interrelationships among alexithymia, meaning in life, and quality of life in older breast cancer patients. Variable-centered methods confirmed that alexithymia significantly negatively impacts quality of life, while meaning in life plays a significant partial mediating role. Additionally, person-centered latent profile analysis identified two distinct subgroups: high alexithymia–low meaning in life and moderate alexithymia–moderate meaning in life, which exhibited notably different outcomes in terms of quality of life. These findings not only extend the application of meaning-construction theory to the field of geriatric oncology but also emphasize the need for targeted interventions to address emotional processing deficits and existential issues. By integrating individual and individual-difference perspectives, this study conducted a nuanced analysis of the psychosocial mechanisms of older breast cancer patients and provided a solid foundation for developing targeted strategies to promote emotional expression, drive meaning reconstruction, and ultimately enhance the quality of life of this vulnerable population.

## Data Availability

The raw data supporting the conclusions of this article will be made available by the authors, without undue reservation.
