# Supplementary material for: The relationship between alexithymia, meaning in life, and quality of life in older breast cancer patients: a cross-sectional study based on variable-centered and person-centered perspectives
Source: Front Med (Lausanne). 2026 Aug 18;13:1708876. doi: 10.3389/fmed.2026.1708876 (PMC13530904; doi:10.3389/fmed.2026.1708876)
Supplement: Supplementary file 1 [file Table_1.DOCX]

# The Relationship between Alexithymia, Meaning in Life, and Quality of Life in Elderly Breast Cancer Patients: A Cross-sectional Study based on Variable-centered and Person-centered Perspectives

# Appendix A

**Univariate analysis of the optimal latent profile**

The results showed that tumor stage (χ²=8.643, p=0.034) and residence (χ²=6.59, p=0.01) were significantly different between the two groups. However, there were no significant differences in gender (χ²=0.584, p=0.445), education (χ²=7.998, p=0.092) and age (χ²=2.205, p=0.332) between the two groups.

| Variables | Items | Moderate alexithymia–moderate meaning in life | High alexithymia–low meaning in life | χ² | p |
| --- | --- | --- | --- | --- | --- |
| Gender | Male | 32 | 7 | 0.584 | 0.445 |
|  | Female | 465 | 73 |  |  |
| Education level | Primary school | 17 | 6 | 7.998 | 0.092 |
|  | Junior high school | 273 | 35 |  |  |
|  | Technical secondary school, high school | 186 | 34 |  |  |
|  | Junior college and above | 21 | 5 |  |  |
| Age | 60-65 years | 234 | 40 | 2.205 | 0.332 |
|  | 66-75 years | 169 | 21 |  |  |
|  | 75 and above | 94 | 19 |  |  |
| Place of residence | Urban | 180 | 41 | 6.590 | 0.01 |
|  | Rural | 317 | 39 |  |  |
| Tumor staging | Stage Ⅰ | 25 | 10 | 8.643 | 0.034 |
|  | Stage Ⅱ | 190 | 33 |  |  |
|  | Stage Ⅲ | 204 | 24 |  |  |
|  | Stage Ⅳ | 78 | 13 |  |  |

Multivariate Logistic regression analysis

The study showed that living in the city was the influencing factor for entering the moderate alexithymia and moderate life meaning group, and the regression coefficient was B= -0.594, P=0.017, OR=0.552, 95%CI was 0.340-0.898, suggesting that compared with rural patients, urban patients were less likely to enter the high alexithymia and low life meaning group.

In terms of tumor stage, taking stage Ⅳ as a reference, the regression coefficient of stage Ⅰ patients was B= -0.990, P=0.042, OR=0.372, 95%CI: 0.143-0.963, suggesting that stage Ⅰ patients were less likely to enter the high alexithymia and low life meaning group than stage Ⅳ patients. However, there was no significant difference between stage Ⅱ (OR=0.861, 95%CI: 0.426-1.739, P=0.677) and stage Ⅲ (OR=1.204, 95%CI: 0.575-2.521, P=0.622) and stage Ⅳ.

| Variable | Items | B | SE | Wald χ² | p | OR | LLCI | ULCI |
| --- | --- | --- | --- | --- | --- | --- | --- | --- |
| Place of residence | Urban | -0.594 | 0.248 | 5.725 | 0.017 | 0.552 | 0.34 | 0.898 |
|  | Rural |  |  |  |  |  |  |  |
| Tumor staging | Stage Ⅰ | -0.990 | 0.486 | 4.153 | 0.042 | 0.372 | 0.143 | 0.963 |
|  | Stage Ⅱ | -0.149 | 0.359 | 0.174 | 0.677 | 0.861 | 0.426 | 1.739 |
|  | Stage Ⅲ | 0.186 | 0.377 | 0.243 | 0.622 | 1.204 | 0.575 | 2.521 |
|  | Stage Ⅳ |  |  |  |  |  |  |  |
